# Long Non-Coding RNAs Can Govern the Antiviral Immune Response Through Interferon-Mediated Mechanisms in Respiratory Tract

**DOI:** 10.3390/v18020231

**Published:** 2026-02-12

**Authors:** Alexey Lozhkov, Alexey Skvortsov, Valeria Kirenskaya, Andrey Vasin

**Affiliations:** 1 Institute of Biomedical Systems and Biotechnology, Peter the Great St. Petersburg Polytechnic University, Polytechnicheskaya St. 29, St. Petersburg 195251, Russia; colbug@mail.ru (A.S.); samsagirlshrek@gmail.com (V.K.);; 2 Smorodintsev Research Institute of Influenza, Prof. Popov St. 15/17, St. Petersburg 197022, Russia

**Keywords:** long non-coding RNAs, Influenza A virus, innate immunity, interferons

## Abstract

Many long non-coding RNAs (lncRNAs) are able to control interferon-dependent innate immune responses and the susceptibility to influenza infection. These lncRNAs are primarily regulated through the RIG-I/IFN-β/IFNAR1 pathway and can be considered as interferon-stimulated genes with either antiviral or proviral functions. In this review we observe the current knowledge of type I and III interferon signaling regulation and discuss the present data on specific lncRNAs, which are involved in the interferon response. The available data on mechanisms of lncRNA induction and action are summarized. Also, the brief overview of genes coding for lncRNAs involved in interferon expression regulation is presented with a focus on the evolutionary conservation of these regulatory molecules. The lncRNAs belong to various classes: antisense, bidirectional, intronic, or intergenic RNAs. Research of lncRNAs is an extremely promising scientific area. Deeper understanding of lncRNA functions may result in the development of new approaches to influenza infection treatment, as well as advanced understanding of the disease pathogenesis. Further bioinformatic analysis of lncRNAs is required to reveal putative common mechanisms of lncRNA action.

## 1. Introduction

Long non-coding RNAs (lncRNAs) are continuous RNA transcripts, longer than 200 nucleotides, that are typically not translated into proteins. Nowadays, lncRNAs are regarded to act as an additional regulatory mechanism that governs multiple biological processes. In a large number of works the involvement of lncRNAs in modulating the immune response against influenza has been demonstrated. According to modern concepts, lncRNAs can take part either in facilitating Influenza A virus (IAV) infection or inhibiting IAV infection; they even can be involved in attenuating viral pathogenicity [1]. A recent study has been published on the role of lncRNAs in immune response against influenza [2]. Among different mechanisms of lncRNA-influenza interplay, we focused on the impact of lncRNAs on interferon (IFN)-dependent antiviral signaling. This review is specifically focused on the IFN-mediated response against influenza and the contribution of lncRNAs to its regulation in human and mouse systems. We discuss the mechanisms of lncRNA induction and action in respiratory epithelial and myeloid cells, as well as analyze the evolutionary conservation of these lncRNAs using current genomic data.

Nevertheless, several examples of IFN-independent lncRNA action can be mentioned. For example, in the recent decade, it was demonstrated that lncRNA IPAN facilitates IAV infection as it stabilizes RNA-dependent RNA polymerase PB1, promoting influenza reproduction [3], IPAN also prevents retinoic acid-inducible gene 1 (RIG-I) and tripartite motif-containing protein 25 (TRIM25)-mediated PB1 degradation [4]; PAAN interacts with influenza PA protein and promotes the assembly of the viral RNA polymerase complex [5]; Lnc-ALOX12 is associated with the PB2 subunit, facilitating the interaction between PB2 and importin-α/β and promoting PB2 nuclear import [6]. On the contrary, Lnc-PINK1-2:5 stimulates expression of thioredoxin interacting protein (TXNIP), which is considered to be an antiviral product [7]. This way, we note that lncRNAs can exert proviral or antiviral action, using mechanisms different from early innate immune response modulation. However, beyond doubt, IFNs have a determining influence on susceptibility to influenza infection and antiviral immune response, and lncRNAs play a significant role in their regulation.

## 2. Methodology

A literature search was conducted in the PubMed database (NCBI, USA) for relevant scientific articles published in the years 2016–2025, up to 15 December 2025. The initial set was generated using the search request “lncRNA” AND “Interferon” AND “Influenza”. The literature search was conducted in English, regardless of the type of publication. The subset was then processed manually, using abstracts in obvious cases and full texts in ambiguous cases. Studies that met the following criteria were included (1): lncRNA-mediated regulation of IFN or interferon-stimulated gene (ISG) expression; (2) lncRNA action in human cells or mouse models; (3) lncRNA expression induction by influenza or pattern-recognition receptor (PRR) agonists; (4) influenza infection. The following exclusion criteria were considered: (1) no relation to IFN or ISG expression; (2) influenza was not studied in the research; (3) reviews, commentaries, or letters; conference proceedings or abstracts; staged trials; or studies without comparative information were ruled out.

## 3. Overview of Type I and Type III IFN Signaling

**Figure 1:**
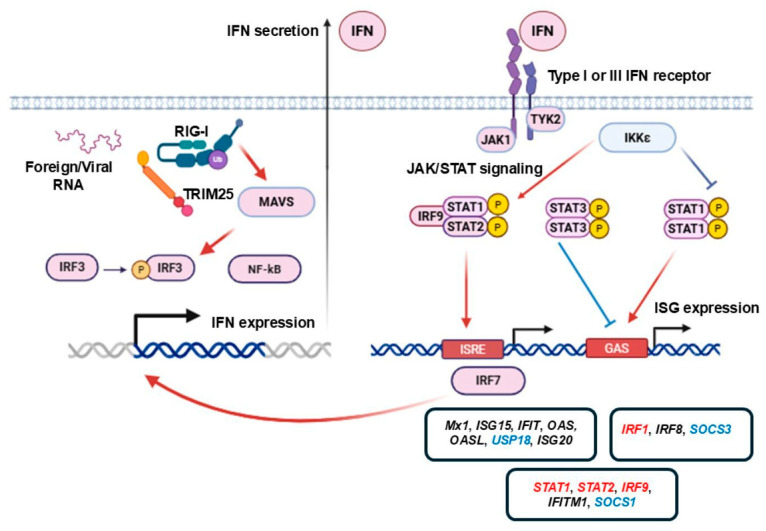
General overview of IFN induction and type I or III IFN signaling. Foreign and viral RNAs induce PRR activation. Cytosolic RNA sensor RIG-I plays significant role in the detection of invading RNA with specific features (5′-diphosphate or 5′-triphosphate group, duplex structure, no ribose 2′-*O*-methylation). TRIM25 stimulates K63-linked RIG-I ubiquitination and activates downstream signaling pathways. Activated multimeric form of RIG-I interacts with its protein adaptor (MAVS), which acts as a scaffold protein, activates IRF3 and NF-κB transcription factors, and induces IFN expression. Type I and III IFNs act in autocrine or paracrine manner, bind to their heterodimeric receptors, and induce JAK/STAT pathways activation. IFN action can result in ISGF3 complex assembly (IRF9/STAT1/STAT2) that induces the expression of ISRE-containing ISGs (Mx1, ISG15, IFIT1-3, OAS1-3, OASL, USP18, ISG20). Also, the expression of IRF7 is upregulated by ISGF3, IRF7 binds to the type I and type III IFN promoters, which results in feedforward loop and IFN expression amplification. Alternatively, GAF complex assembly (STAT1 homodimer) induces GAS-containing gene expression (IRF1, IRF8, SOCS3). Several ISGs possess both ISRE and GAS regulatory sequences (STAT1, STAT2, IRF9, IFITM1, SOCS1). Type I IFN-induced STAT3 homodimer inhibits STAT1-mediated antiviral response. IKK-ε also suppresses STAT1 homodimer formation, facilitating ISGF3 assembly and promoting antiviral response. Genes that attenuate JAK/STAT signaling are indicated in blue (SOCS1, SOCS3, USP18), and genes promoting JAK/STAT signaling are depicted in red (IRF1, IRF9, STAT1, STAT2). Red arrows represent activation; blue arrows–inhibition; black–secretion/gene expression; light blue ellipses–regulatory factors; red ellipses–IFN expression upregulating factors; P–phosphorylation; Ub–ubiquitination.

Different pathogen-associated molecular patterns (PAMPs) induce the activation of the innate immune response. Cells utilize various PRRs to detect penetrating viral particles, including endosomal Toll-like receptors (TLRs) and cytosolic RIG-I-like receptors, Nod-like receptors, and DNA sensors. In the context of RNA viruses, RIG-I-like receptors are of particular interest; they are represented by RIG-I and melanoma differentiation-associated protein 5 (MDA5). Cytosolic RIG-I and MDA5 bind to foreign RNAs. They contain two *N*-terminal domains known as caspase activation and recruitment domains (CARDs), which are necessary for further immune response activation, as well as a central helicase domain and a *C*-terminal domain, which detect foreign RNA molecules [8,9]. In resting conditions, RIG-I and MDA5 are present in monomeric form, in auto-inhibited conformation [9]. Penetration of foreign RNAs promotes conformational changes in the RNA sensors and their oligomerization. It is considered that RIG-I binds to RNA in its monomeric form; however, RIG-I signaling requires RIG-I oligomers that are stabilized by non-degradative K63-polyubiquitin chains [9]. The activated multimeric forms of RIG-I or MDA5 are able to interact through homotypic CARD–CARD interactions with the mitochondrial antiviral signaling protein (MAVS) adapter, which is anchored to the mitochondrial or peroxisome membrane. The adapter acts as a scaffold protein and activates TANK-binding kinase-1 (TBK-1) and Inhibitor of NF-κB kinase-ε (IKK-ε), which leads to further activation of the transcription factors Interferon regulatory factor 3 (IRF3) and nuclear factor κB (NF-κB). These transcription factors induce IFN production (Figure 1) [8,9,10].

RIG-I is involved in immune responses against various types of viruses, including dsDNA (*Herpesviridae*, *Adenoviridae*), dsRNA (*Reoviridae*), plus ssRNA (*Picornaviridae*, *Flaviviridae*, *Coronaviridae*), and minus ssRNA (*Orthomyxoviridae*, *Paramyxoviridae*, *Filoviridae*) viruses. Viral RNAs are conventional RIG-I agonists and are characterized by a combination of specific features (5′-diphosphate or 5′-triphosphate group, duplex structure, no ribose 2′-*O*-methylation) [9]. RIG-I modifications with ubiquitin modulate its antiviral activity. K48-linked ubiquitination leads to RIG-I proteasomal degradation, whereas K63-linked modification facilitates the innate response [11]. TRIM25 is a well-known E3 ubiquitin ligase that interacts with the CARD domain and stimulates K63-linked ubiquitination at the Lys172 residue. Other ubiquitin ligases, such as Riplet (RNF135 or REUL), are also essential for RIG-I ubiquitination [12]. After TRIM25-mediated modification, RIG-I forms tetramers that transfer to mitochondria for MAVS activation and IFN expression [8,11]. Moreover, RIG-I and TRIM25 cooperatively evoke influenza PB2 degradation in a signaling-independent manner [4].

In response to a viral infection signal, cells start producing IFN-β, which acts in an autocrine manner and stimulates the production of other type I IFNs. This IFN-β-dependent IFN induction is known as priming [13]. Beta IFN expression requires the activation of NF-κB and IRF3 (Figure 1). In a resting state, NF-κB and IRF3 are anchored in the cell membrane. After the detection of viral pathogens, NF-κB and IRF3 translocate to the nucleus. Together with the c-jun/ATF-2 heterodimer, NF-κB and IRF3 are involved in enhanceosome assembly on the IFNB promoter. IRF3 is considered to be crucial for the formation of the protein complex. Enhanceosome formation facilitates the recruitment of the CREB-binding protein and gene expression [13]. IFN-β acts autocrinally or paracrinally to induce the expression of IRF7 that binds to the type I and type III IFN promoters and stimulates the expression of the respective genes [14].

IFNs are important mediators of the antiviral immune response. Nowadays, three types of IFNs are known. In the context of antiviral response, type I and type III IFNs are of particular interest. Cell-invading viruses or dsRNAs induce considerable expression of these types of IFNs. IFNs-α, IFN-β, and IFN-ω are type I IFNs that exhibit distinct antiviral activity [15]. IFN-β and IFN-ω are products of the IFNB and IFNW genes, respectively; at least 13 genes encoding different subtypes of alpha IFNs (forming a compact gene cluster) have been identified [16]. Type I IFN encoding genes are located on chromosome 9 (here and below, gene count, chromosome locations, and amino acid positions are given for human genes and proteins, unless specified explicitly). Type I IFNs act through the heterodimer IFN-α receptor (IFNAR), which consists of IFNAR1 and IFNAR2 subunits and is expressed ubiquitously [16]. In the early XXI century, type III IFNs, also known as IFNs-λ, were discovered. IFN-III contains four subtypes: IFN-λ1, IFN-λ2, IFN-λ3, and IFN-λ4. Type III IFNs are encoded by IFNL genes, which are located on chromosome 19 [16]. Lambda IFNs act through the heterodimeric IFN-λ receptor (IFNLR) that consists of IL10R2 and IFNLR1 subunits, and its expression is limited to certain cell types. High IFNLR1 expression has been found in the lungs, intestines, liver, and upper epidermis. Expression of IFNLR1 is mainly restricted to epithelial cells, keratinocytes, differentiated dendritic cells, and hepatocytes [17]. The exon-intron structure of type I and type III IFN genes also varies greatly; IFNA, IFNB, and IFNW mRNAs consist of a single exon, whereas IFNL mRNAs are characterized by a multi-exon structure [16]. In turn, in response to foreign antigens or mitogens, type II IFNs are mainly produced by T lymphocytes and natural killer cells. IFN-γ is the sole member of the type II IFN family; it is encoded by the IFNG gene, which is located on chromosome 12. The receptor for IFN-γ is built of two IFNGR1 and two IFNGR2 subunits and mediates a broad spectrum of immune responses to non-viral pathogens [18]. Interestingly, the receptors for each type of IFN consist of a high-affinity subunit (IFNAR2 for type I IFNs, IFNGR1 for IFN-γ, and IFNLR1 for IFNs-λ) and a low-affinity subunit (IFNAR1, IFNGR2, and IL10R2, respectively) [19].

IFNs activate the antiviral defense mechanisms, preparing cells for potential viral infection, and induce the expression of ISGs. Type I and type III IFNs actions induce dimerization of receptor subunits and the activation of tyrosine kinases: Janus kinase-1 (JAK1) and tyrosine kinase-2 (Tyk2). IFN-γ activates Janus kinases JAK1 and JAK2. Tyrosine kinases phosphorylate STAT family transcription factors (signal transducer and activator of transcription). Prior to activation, the cytoplasmic tail of the low-affinity subunit is associated with Tyk2, and the high-affinity subunit is associated with JAK1 (Figure 1). IFN-induced subunit dimerization causes a conformational change; Tyk2 phosphorylates the corresponding low-affinity subunit, creating a docking site for STAT2. Tyk2 then phosphorylates STAT2 on tyrosine 690, and STAT1 is phosphorylated by JAK1 on tyrosine 701 (Figure 1) [13]. Phosphorylation of STAT1 and its dimerization with STAT2 create a novel nuclear localization signal, whereas STAT2 phosphorylation inhibits constitutive nuclear export of STAT2 [13].

Phosphorylated STAT1 can form a homodimer (known as γ-activated factor, GAF) (Figure 1). GAF assembly can be induced by either IFN-α/β, IFN-λ (less potent activation), or IFN-γ. GAF upregulates the expression of γ-activated sequence (GAS)-containing proinflammatory genes. GAS-elements are present in the promoters of many ISGs (Figure 1). Alternatively, type I IFNs can induce STAT3 homodimer assembly. The latter also binds to GAS-elements; however, it suppresses proinflammatory gene expression [20]. Thus, STAT3 can be regarded as a negative regulator of the STAT1-mediated antiviral response downstream of IFNAR [21].

Type I and type III IFNs also evoke interferon-stimulated gene factor 3 (ISGF3) formation, consisting of IRF9 and phosphorylated forms of STAT1 (pTyr701) and STAT2 (pTyr690). The action of IFNs induces the recruitment of CREB-binding protein to the IFNAR2 subunit and catalyzes its acetylation, which creates a docking site for IRF9. In turn, IRF9 gets acetylated, as do the receptor-bound STAT1 and STAT2. Acetylation of IRF9 is required for DNA binding, and acetylation of the STAT factors may aid ISGF3 complex assembly (Figure 1) [13]. IRF9 has a strong nuclear localization signal that enables ISGF3 factor import to the nucleus. On the contrary, the nuclear export signal of dephosphorylated STAT2 shuttles the complex back to the cytoplasm. Alternatively, a complex containing IRF9 and phosphorylated STAT2 dimer is known. ISGF3 (IRF9/STAT1/STAT2) and IRF9/STAT2/STAT2 factors activate the expression of interferon-stimulated response element (ISRE)-containing genes (Figure 1). IKK-ε phosphorylates STAT1, which suppresses STAT1 homodimer formation, facilitating ISGF3 assembly and promoting antiviral response. The absence of IKK-ε prevents ISGF3 complex formation and the expression of ISRE-dependent ISGs in mouse models [22]. Moreover, IRF1 can upregulate ISG expression in response to IFN action directly binding the ISRE or IRF-responsive element (IRE) [18]. Several ISGs encode canonical antiviral factors, including myxovirus resistance protein (MxA), 2′-5′-oligoadenylate synthetase (OAS), protein kinase R (PKR), interferon-induced proteins with tetratricopeptide repeats (IFIT), interferon-induced transmembrane protein (IFITM), and ISG15. Notably, RNA sensors RIG-I and MDA5 are also upregulated by IFN action [23,24,25]. ISGs Mx1, ISG15, IFIT1-3, OAS1-3, OASL, USP18, and ISG20 contain only the ISRE regulatory sequence (AGTTTCN_2_TTTCN); IRF1, IRF8, and suppressor of cytokine signaling 3 (SOCS3) contain the GAS element (TTCN_2–4_GAA); and the STAT1, STAT2, IRF9, IFITM1, and SOCS1 genes have both regulatory sequences. The IRF7 gene contains IRE and ISRE promoters [26], and its expression leads to a feedforward loop and facilitates IFN production [20]. Promyelocytic leukemia zinc finger protein (PLZF) is another transcription factor that is implicated in ISG expression regulation [20].

Type I and type III IFN expression is tightly regulated. Transcription factors STAT1 and IRF9 are themselves ISGs, providing IFN-dependent autocrine positive feedback. Under homeostatic conditions, a low IFNB level is able to maintain high basal STAT1 and IRF9 expression. Basal IFNAR signaling is also activated through immunoreceptor tyrosine-based activation motif (ITAM)-dependent mechanism [20]. Elevated STAT1 and IRF9 expression and STAT phosphorylation cause type I and III IFN signaling amplification. Proinflammatory cytokines such as interleukin 6 (IL6) and tumor necrosis factor-α (TNF-α) can also promote the expression of these transcription factors [20,27,28].

There are several mechanisms of negative regulation of type I and type III IFNs. IFN-α/β signaling can be attenuated at the level of receptor expression. After IFN-α or IFN-β binding, the ternary complex can undergo clathrin-mediated endocytosis [19]. IL1β action limits cellular IFN responsiveness, inducing IFNAR internalization and degradation. Moreover, strong activation of ITAM-containing receptors suppresses IFNAR signaling via protein-tyrosine phosphatase 2 (SHP2) action [20]. Also, protein-tyrosine phosphatase 1B (PTP1B) binds to the IFNAR1 subunit and regulates AP-2-dependent endocytosis. The absence of PTP1B is associated with increased activity of the JAK/STAT pathways and excessive IFN production [19].

SOCS1, SOCS3, and ubiquitin-specific peptidase 18 (USP18, also known as UBP43) are ISGs that inhibit JAK/STAT signaling, providing a negative feedback loop in the IFN signaling [18,29]. USP18 is an ISG15-specific protease that deconjugates ISG15 with the target protein and reduces the level of ISG15-conjugated proteins. In addition, USP18 can bind to the IFNAR2 subunit and compete with JAK1 kinase, leading to a decrease in JAK/STAT-dependent signaling activity. Knockout of Usp18 in mice led to hypersensitivity to type I IFN action [30,31]. SOCS proteins are considered to be the major inhibitors of JAK/STAT-dependent signaling pathways. SOCS1 and SOCS3 proteins compete with STATs for binding to receptor subunits and act as a kinase pseudo-substrate, also blocking JAK activity [20,32].

The expression of IFNs and ISGs can be regulated at the transcriptional level. Under normal circumstances, unmodified histone octamer cores strongly bind to DNA, hindering gene expression. Also, forkhead box protein O3 (FOXO3) is known to repress the IFN response [20]. In turn, IFN-dependent induction of ISGs requires chromatin remodeling and post-translational modification of histones. STAT1, STAT2, and IRF family factors bind to their promoters or enhancers, facilitating the recruitment of nucleosome-remodeling enzymes and histone acetyltransferases. Also, type I IFNs induce H2B ubiquitylation. The ISGF3 complex recruits lysine acetyltransferase (CREB-binding protein); the BRG1 chromatin remodeling factor; bromodomain-containing protein (BRD4) that interacts with acetylated lysine residues; and the multi-subunit Mediator co-activator complex for RNA polymerase II-dependent transcription. Pausing of RNA polymerase II at inducible genes that encode the IFN pathway dampens basal type I IFN signaling [33]. Moreover, epigenetic factors can predispose IFN response. The expression of IFNs and ISGs is inversely correlated with di-methylation of histone H3 at lysine 9 (H3K9me2). In turn, inhibition of lysine methyltransferase G9a, which is essential for these histone modifications, resulted in phenotypic conversion of fibroblasts into highly potent IFN-producing cells [34].

This way, we reviewed the most indispensable features of type I and type III IFN-dependent signaling. IFN signaling is also under tight control by RNA regulators [1,20]. The main part of the work is devoted to the mechanisms of lncRNA-mediated modulation of the signaling.

## 4. LcnRNAs Can Modulate Systemic IFN Response

The influence of lncRNAs on IFN-mediated signaling is implemented at several levels at once. LncRNAs can regulate transcription, splicing, nucleic acid degradation and serve as decoys, signals, guides, or scaffolds. As scaffolds, lncRNAs act on chromatin to modulate transcription by altering histone modifications, nuclear structures, or signaling complexes [11]. LncRNAs are able to modulate cytosolic RNA sensor activity [35,36], modulate the expression of IFNs and multiple ISGs [37,38,39,40,41], act as a competing endogenous RNA (ceRNA) to regulate the expression of a specific target mRNA transcript [42,43,44], affect JAK/STAT signaling [44,45,46,47], or even encode small proteins that are able to regulate the susceptibility to influenza infection [48]. In reverse, it has been common knowledge for over ten years that IFNs are able to upregulate lncRNA expression [49,50].

The research of lncRNAs is a rapidly developing field, so the naming of the species in literature is associated with a certain degree of ambiguity. In the present work we will mostly focus on human lncRNAs or their proved mouse orthologs and denominate them according to their names in human genome/transcriptome databases whenever possible. Additional information on the considered lncRNAs and the respective genes may be found in Table 1 and Table 2.

LncRNAs were shown to modulate IFN-dependent antiviral signaling in myeloid cells (primarily macrophages) [11,51,52,53]. LncRNA CHROMR or CARINH knockdown facilitates IAV infection [51,52], whereas lncNSPL upregulation promotes IAV reproduction [53]. LncRNA IVRPIE is mainly expressed in blood immune cells as well as in lung cells and promotes IFN-β and ISG expression. The latter lncRNA will be discussed in the next chapters [37].

**Figure 2:**
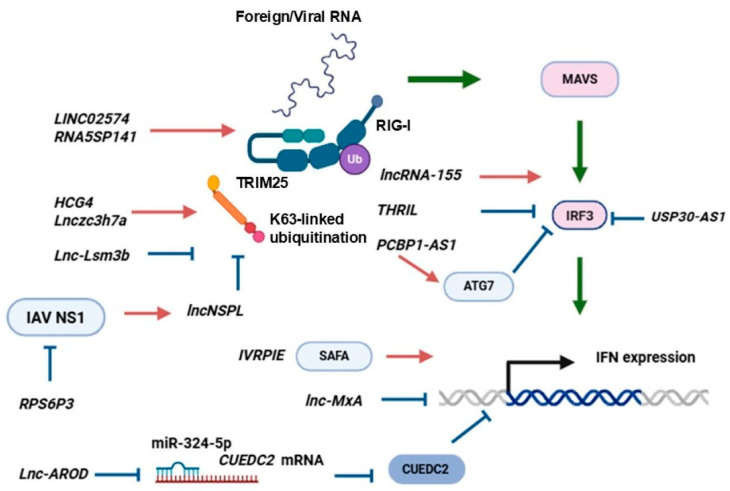
The influence of lncRNAs on RIG-I/MAVS-dependent IFN induction. Foreign and viral RNAs induce the activation of RIG-I/MAVS pathways and IFN expression. TRIM25 stimulates K63-linked ubiquitination and facilitates the innate immune response. In epithelial cells and macrophages, lncRNAs are able to modulate IFN expression at several levels at once: they regulate RIG-I production and activation (LINC02574, RNA5SP141); TRIM25-mediated RIG-I ubiquitination (HCG4, Lnczc3h7a, Lnc-Lsm3b, LncNSPL, RPS6P3); IRF3 activation (LncRNA-155, THRIL, PCBP1-AS1, USP30-AS1); IFN promoter activation (Lnc-MxA, Lnc-AROD); or histone-modifying factor action (IVRPIE). Red arrows represent the activation of IFN upregulating pathways by lncRNAs; blue arrows represent inhibitory lncRNA action; green arrows represent signaling events causing IFN expression; light blue ellipses represent proteins/factors; a combination of a lncRNA and a protein (for example, IVRPIE and SAFA) indicates their direct interaction; miRNAs are depicted in blue, mRNAs are depicted in red, and Ub represent ubiquitination.

Several lncRNAs are able to affect the immune response at the level of RIG-I activation (Figure 2). For instance, in murine macrophages and immune cells, Lnczc3h7a binds to both TRIM25 and activated RIG-I, serving as a molecular scaffold for stabilization of the RIG-I-TRIM25 complex at the early stage of viral infection and strengthening RIG-I-mediated IFN expression. The lncRNA was found to be upregulated in response to various PRR ligands, type I IFNs, and RNA viruses. *In vivo* experiments showed distinct Lnczc3h7a antiviral action against vesicular stomatitis virus (VSV). In response to IAV, Lnczc3h7a^−/−^ mice produced less type I IFNs and IL6 in serum than their littermates with the functional gene [11]. VSV, Sendai virus (SeV), dsRNA, and IFNs also stimulated the expression of another lncRNA, lnc-Lsm3b, in murine macrophages that acted as a negative regulator of RIG-I-dependent signaling, IRF3 and NF-κB activation, as well as of IFN production. The molecular mechanism of lnc-Lsm3b action is based on disturbed TRIM25-mediated K63-linked ubiquitination of RIG-I upon viral infection that inhibits MAVS-mediated pathways. Also, it is suggested that lnc-Lsm3b hinders the conformational shift of RIG-I, preventing downstream signaling and type I IFN production (Figure 2). This way, lnc-Lsm3b expression is a negative feedback mechanism that downregulates late IFN and ISG expression. However, the role of Lnczc3h7a and lnc-Lsm3b in the context of influenza infection needs to be determined [11,23].

In IAV-infected human monocytes, RIG-I upregulation coincides with elevated lncNSPL level. This lncRNA was shown to directly bind to the cytosolic sensor, suppressing the interaction between RIG-I and TRIM25. Mechanistically, lncNSPL disturbed the TRIM25-mediated K63-linked ubiquitination of RIG-I in IAV-infected cells (Figure 2). The SPRY domain of TRIM25 binds to the exposed CARD domains of RIG-I to mediate ubiquitination. Probably, lncNSPL binds to RIG-I helicase and *C*-terminal domains via a stem-loop RNA structure that may restrict the cytosolic sensor protein conformation shift, especially in the CARD domain, lowering RIG-I’s ability to interact with TRIM25. Influenza utilizes non-structural NS1 protein to evade the immune response. It was shown that viral NS1 is able to stimulate lncNSPL expression. In IAV-infected THP-1 cells, lncNSPL knockdown resulted in elevated proinflammatory cytokines (IFN-β, IL6, and IL1β) and inhibited IAV replication, and proviral lncNSPL action is mediated by attenuation of NF-κB and IFN-β promoter activation and weakened IRF3 and p65 phosphorylation. Corresponding results were achieved in mouse experiments. Intranasal lncNSP administration led to higher susceptibility of mice to lethal influenza infection (increased lethality and viral load, severe pathological changes and elevated apoptosis in lungs, and decreased serum and lung proinflammatory cytokines). This way, lncNSPL can be considered as an NS1-induced innate immune response inhibitor that operates at least in white blood cells [53].

CHROMR and CARINH exhibit antiviral action. CHROMR is upregulated in white blood cells of patients with IAV or SARS-CoV-2. CHROMR-deficient macrophages were characterized by repressed IFN signaling and weakened IAV- or Polyinosinic:polycytidylic acid (Poly(I:C))-induced ISGs and chemokines; meanwhile, CHROMR overexpression had the opposite effect. IRFs are indispensable transcription factors for IFN induction, and their tight regulation may determine the susceptibility to infection [52,54]. It was demonstrated that CHROMR sequesters the IRF2/IRF2BP2 complex that acts as an inhibitor of the expression of ISGs. The complex antagonizes IRF1-mediated transcriptional activation and hinders ISG transcription [52]. This way, CHROMR is able to block the action of the IRF1 inhibitor and stimulate the IFN response. IRF2 expression was also demonstrated to be downregulated by miR-302. MiR-302b-3p stimulated the activation of IFN-β, the NF-κB promoter, and ISRE elements; it also facilitated IAV-induced expression of antiviral protein-coding genes and lncRNAs. It was revealed that miR-302 cluster members are able to bind to the 3′-UTR of IRFs (mainly IRF1, IRF2, and IRF9) and upregulate an antisense lncRNA, IRF1AS. It can function as an enhancer cluster that orchestrates and cis-regulates the transcription of IRF1, thereby rapidly amplifying the antiviral immune response [54].

A cis-acting lncRNA, CARINH, also regulates the expression of its antisense gene, IRF1. CARINH and IRF1 are coordinately increased in the circulation of patients infected with IAV, human metapneumovirus, or SARS-CoV-2. CARINH depletion attenuated IRF1 production, IRF-driven transcription, and IFN-β, IFN-γ, and IFN-λ secretion in Poly(I:C)-treated THP1 cells. A functional CARINH ortholog was found in the mouse genome. CARINH knockout reduced antiviral immunity and increased viral burden upon challenge with IAV, although there were no significant differences in mouse morbidity or mortality [51].

**Figure 3:**
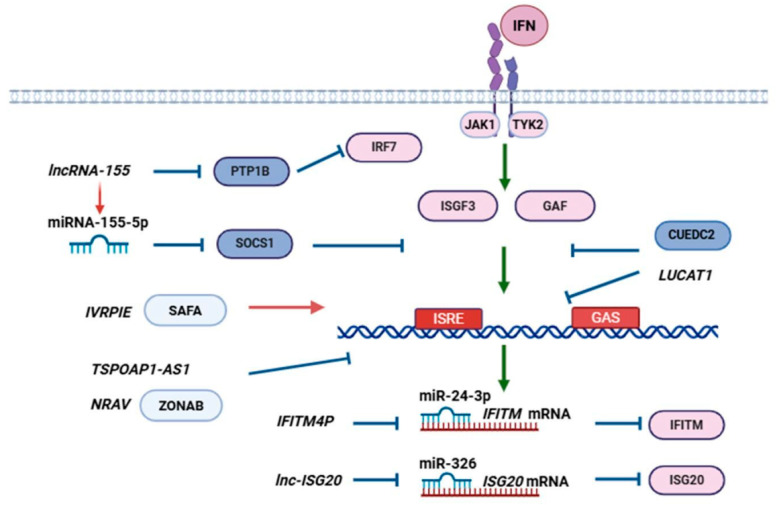
LncRNA action downstream of IFN signaling. Type I and III IFNs stimulate JAK/STAT pathways that induce the expression of ISGs. LncRNAs can affect ISG transcription (IVRPIE, NRAV, TSPOAP1-AS1); sponge miRNAs that downregulate ISG translation (Lnc-ISG20, IFITM4P); and attenuate STAT1-mediated gene expression (LUCAT1). LncRNA-155 and its product miR-155 have distinct roles in innate immunity regulation; miR-155 is considered to attenuate SOCS1-dependent JAK/STAT pathways inhibition, while LncRNA-155 stimulates INFB expression and blocks PTP1B action. Red arrows represent the activation of ISG upregulating pathways by lncRNAs; blue arrows represent inhibitory lncRNA action; green arrows represent signaling events causing ISG expression; light blue ellipses represent lncRNA interacting proteins/factors; red ellipses represent ISG/IFN upregulating proteins; dark blue ellipses—ISG inhibiting factors; miRNAs are depicted in blue; mRNAs are depicted in red and Ub represent ubiquitination.

In primary monocyte-derived human dendritic cells, lncRNA LUCAT1 is upregulated in response to lipopolysaccharide (LPS) stimulation, HSV-1, or IAV infection. Also, LUCAT1 expression was increased in LPS-treated primary human CD14^+^ monocytes, dendritic cells, and macrophages. LUCAT1 deficiency is associated with ISG upregulation, whereas its overexpression has the opposite effect. It was demonstrated that LUCAT1 was a chromatin-associated lncRNA that interacted with STAT1 in the nucleus and hindered the transcription of ISGs (CCL5, IP10, IFIT1, RSAD2) (Figure 3) [55].

Additionally governing the immune response in peripheral blood cells, IFNs are the first defensive barrier of respiratory epithelial cells, where lncRNA-mediated IFN regulation also plays the determining role.

## 5. The Diversity of IFN-Regulating lncRNAs in Respiratory Epithelial Cells

Airway epithelial cells are primary targets for respiratory viruses. They are the first protective defensive barrier of our organism. The interaction between airway mucosa and invading viral pathogens largely determines the course of infection and its outcome. Our knowledge about lncRNA-mediated immune regulation in respiratory epithelial cells has increased significantly. Most likely, in the nearest years we will learn about new examples of such regulation. Nevertheless, it is already possible to trace the basic features of lncRNA induction and functioning.

### 5.1. Expression Features

In response to the signal of viral penetration, the expression of lncRNAs occurs that allows adjustment of the immune response. Generally, the considered lncRNAs were shown to be upregulated in response to infection by RNA-containing viruses, poly(I:C), or IFN-β treatment in a RIG-I and NF-κB-dependent manner (Table 1). Poly (I:C) RNA is a conventional TLR3 and MDA5 ligand and stimulates multiple lncRNAs [37]. Several lncRNAs are shown to be dependent on JAK/STAT signaling and regarded to be ISGs [47,56]; however, IFNs can also downregulate lncRNA expression [39]. In turn, IVRPIE expression is considered to be IFN-β- and NF-κB-independent [37]. LPS or IL6 stimulation did not affect the considered lncRNA levels (Table 1). RNA viruses such as IAV, SeV, and VSV are primarily recognized by RIG-I and evoke lncRNA expression. VILMIR expression can be upregulated by respiratory syncytial virus (RSV) or SARS-CoV-2 [41]; it is also increased in immune cells of bronchoalveolar lavage fluid samples of COVID-19 patients [41]. The determination of lncRNA expression in human lung tissue samples is of particular interest. LncRNAs ADAM2-2, SIMALR, LINC02865, LGALS17A, and LGALS14 were among the most highly upregulated transcripts in IAV infection in ex vivo cultured human lung tissue explants from patients with emphysema [57].

Viral RNAs with 5′-triphosphate termini induced robust lncRNA expression, while CIAP (calf intestine alkaline phosphatase) treatment of viral RNAs resulted in inhibited lncRNA-155 and IFITM4P expression [42,45]. Similarly, IAV RNA evoked PCBP1-AS1 and LINC02574 expression, while total RNA from intact cells did not affect the lncRNA levels [48,58]. These results underlie the role of cytosolic RNA sensors in lncRNA expression. It was noted that in RIG-I-deficient cells the induction of ISR, lncRNA-155, PCBP1-AS1, LINC02574, and IFITM4P expression by IAV was blocked [42,48,58,59]. Moreover, RIG-I-dependent expression of the lncRNAs was confirmed in C57BL/6 mice [45,59]. This way, it is suggested that lncRNAs are primarily regulated through the RIG-I/IFN-β/IFNAR1 pathway and can be considered as ISGs with either antiviral or proviral functions [36,39,41,42,45,48,56,58,59].

**Table 1:** Features of expression and the mechanism of action of several LncRNAs that are regulated by IAV infection in respiratory epithelial cells.

lncRNA	lncRNA-Expressing Cell Line or Tissue	lncRNA Modulating Agonist or Virus	Expression Features of lncRNA	Molecular Mechanism of lncRNA Action	Refs.
HCG4	A549	Upregulated: Swine influenza virus		HCG4 regulates K63-linked RIG-I ubiquitination, activating RIG-I-dependent pathway and IFN-β production	[35]
IFITM4P	A549, 293T, Huh7, K562, and HeLa;	Upregulated: IFN-β, Poly (I:C) RNA, IAV, SeV, MDRV, HSV-1 No change: PRV	RIG-I silencing blocked IFITM4P expression; IFNAR1 KO decreased IFITM4P expression	Upregulates IFITM expression by acting as ceRNA of miR-24-3p and increasing the stability of IFITM mRNAs	[42]
ISR	A549, 293T cells; Mice NIH/3T3, RAW264.7, and 4T1 cells; Lungs of C57BL/6 mice	Upregulated: IFN-β, Poly (I:C) RNA, IAV, SeV, HSV, PRV; No change: LPS	RIG-I KO suppresses ISR expression in A549 cells and mouse lungs; TLR3 and MDA5 knockdown–no influence on ISR level in A549 cells IFNAR1 KO decreased ISR level in mouse lungs	-	[59]
IVRPIE	A549, BEAS-2B cells	Upregulated: Poly (I:C) RNA, IAV, SeV, VSV No change: IFN-β, RSV, AdV		Enhances IFN-β and ISGs expression via hnRNPU-mediated histone modifications;	[37]
LINC02574	A549, 293T	Upregulated: IFN-β, IFN-λ1, Poly (I:C) RNA, IAV (including H9N2), IAV viral RNA No change: IL6, LPS	RIG-I and IFNAR1 KO dampened LINC02574 expression; MDA5 and TLR3–no influence	LINC02574 KD decreases IAV-induced PRR expression (RIG-I, MDA5, TRL3), attenuates STAT1 and IRF3 phosphorylation, and dampens IFN (IFNB, IFNL1, IFNL2) and ISG expression	[58]
lnc-ISG20	A549, 293T cells	Upregulated: IFN-β, Poly (I:C) RNA, IAV, SeV		Upregulates ISG20 level; Acts as ceRNA by reducing miR-326 binding to the ISG20 3′ UTR	[43]
lncRNA-155	A549, 293T cells; Mice NIH/3T3, LLC, and RAW 264.7 cells; Lungs of C57BL/6J mice	Upregulated: IFN-β, Poly (I:C) RNA, IAV viral RNA; IAV, SeV, MDRV; No change: HSV	RIG-I KO prevents lncRNA-155 expression in A549 cells and mouse lungs; TLR3, IRF3, IRF7, and STAT1 knockdown suppresses lncRNA-155 in A549 cells; MDA5 knockdown–no influence on lncRNA-155 level	Enhances STAT1 activation, Inhibits PTP1B, which leads to IFN and ISG upregulation LncRNA-155 KO results in attenuated STAT1, IRF3 phosphorylation, and lowered IFN-β expression	[45]
RPS6P3	A549, 293T	Upregulated: IFN-β, Poly (I:C) RNA, IAV, SeV	IFNAR1 KO decreased RPS6P3 expression	Prevents NS1-mediated inhibition of IFN-β expression; Interacts with NP and prevents its self-oligomerization	[36]
USP30-AS1	Human primary alveolar epithelial cells, A549, Calu-3	Upregulated: IFN-α, IFN-β, IFN-γ, IAV	Contains two ISRE-like motifs (ACTTTCATTTTTA) in the 5′-region;		[56]
			JAK/STAT-dependent	Modulates prohibitin 1 stability, facilitates prohibitin 1-IRF3 interaction, and blocks IRF3 translocation into the nucleus	[47]
GAPLINC	A549 Mouse lungs	Downregulated: IAV	Downregulated by NF-κB signaling; ATG7-dependent expression, possibly via NF-κB pathway attenuation;	Acts downstream of ATG7, inhibits IRF3 phosphorylation and IFN production; GAPLINC silencing blocks ATG7-mediated upregulation of IAV replication	[60]
lnc-AROD	A549	Downregulated: IAV, SeV, VSV No change: HSV		lnc-AROD interacts with miR-324-5p, leading to decreased binding of miR-324-5p to CUEDC2 and enhanced CUEDC2 expression	[44]
Lnc-MxA	A549	Upregulated: IFN-β, Poly (I:C) RNA, IAV, SeV		Inhibits the activation of the IFN-β transcription and hinders IRF3 and NF-κB binding to the IFN-β promoter	[38]
NRAV	A549, HeLa, K562, 293T, 7721, 7703, and 7402 cells	Downregulated: IAV, SeV, MDRV, HSV No change: LPS		Downregulates ISGs (IFITM3, MxA) initial transcription through affecting histone modification	[61]
PCBP1-AS1	A549	Upregulated: IFN-β, IFN-λ1, Poly (I:C) RNA, IAV viral RNA; IAV, SeV, MDRV, HSV-1, PRV No change: IL6, LPS	RIG-I, MDA5, or MAVS KO attenuated PCBP1-AS1 expression; IFNAR1 silencing negated IFN-β-induced PCBP1-AS1 upregulation	PCBP1-AS1-encoded peptide PESP promotes IAV-autophagy via ATG7 upregulation	[48]
PSMB8-AS1	A549, HEK293, H441	Upregulated: IFN-β, IAV			[62]
THRIL	A549	Downregulated: IFN-β, IFN-λ1, Poly (I:C) RNA, IAV, SeV, PRV No change: LPS	IFNAR1 KO prevents IAV- or IFN-β-induced THRIL downregulation	Attenuates IRF3 phosphorylation, IFNs, and ISGs expression	[39]
TSPOAP1-AS1	A549, Caco-2, and SH-SY5Y cells;	Upregulated: Poly (I:C) RNA, IAV No change: LPS		Inhibits IFN-β transcription and dampens ISRE promoter activation	[40]
VILMIR	A549, BEAS-2B, NTBE, NHBE, Huh7	Upregulated: IFN-β, IAV, RSV, SARS-CoV-2	VILMIR is upregulated by IFN-β in a dose- and time-dependent manner; IFNAR1 KO blocked VILMIR expression	Contains the 5′-binding site of RELB (subunit of NF-κB), IRF1, STAT1, STAT2, and STAT3	[41]

Refs.–References.

### 5.2. LncRNAs That Exhibit Antiviral Action

Several lncRNAs exhibit robust antiviral action, as their overexpression suppresses influenza reproduction and was shown to dampen expression of almost all of the IAV genes: nucleoprotein (NP), hemagglutinin (HA), matrix protein 1 (M1), polymerase (PA, PB1, PB2), non-structural protein 1 (NS1). Conversely, the lncRNAs silencing facilitates viral growth [35,36,37,42,43,45,47,58,59].

**LncRNAs can affect PRR activation.** LncRNA HCG4 affects antiviral response at the level of RNA-sensor activation. HCG4 was found to repress swine IAV (strain A/swine/Shanghai/3/2014 H1N1) replication in HCG4-overexpressing human cells, while HCG4 knockdown strengthened viral NP expression and contributed to the increase in viral titer. In HCG4-overexpressing cells, IAV infection resulted in elevated IFNB expression and RIG-I production, as well as IRF3, STAT1, and STAT2 phosphorylation compared to intact IAV-infected cells. HCG4 silencing fully negated these effects. It revealed that HCG4 was a positive mediator for RIG-I signaling that promoted K63-linked ubiquitination, stimulated IFN-β production, and downstream ISG activation (Table 1) [35]. LINC02574 also blocks influenza infection; LINC02574 knockdown leads to impaired PRR expression (RIG-I, MDA5, and TLR3), IRF3 and STAT1 phosphorylation, and lowered expression of type I and type III IFNs and multiple ISGs (IFI, IFITM1, IFITM3, ISG15, MxA, and OAS1). Thus, LINC02574 may act as a switch to activate antiviral immune responses by regulating PRR signaling; however, the precise molecular mechanism of this lncRNA action is not clear [58]. Influenza developed multiple strategies to evade the innate immune response. As it was discussed in the previous work [53], one of the strategies is the inhibition of the RIG-I-mediated response. Viral NS1 protein hinders TRIM25 and Riplet-mediated RIG-I ubiquitination, which dampens the IFN-mediated immune response. NS1 binds to the coiled-coil domain of TRIM25, inhibiting its ubiquitin ligase activity [12]. Moreover, the NS1 protein of the “Spanish flu” (strain A/Brevig Mission/1918 H1N1) is able to directly interact with the CARD domain of RIG-I and hinders its ubiquitination [63]. LcnRNAs can facilitate innate response activation, blocking NS1 action. Thus, RPS6P3 indirectly stimulates IFN-β as it antagonizes NS1 activity and reinforces IFNB expression through blocking interaction of NS1 with RIG-I and TRIM25 along with restoring RIG-I ubiquitination (Figure 2). Also, RPS6P3 is able to interact with influenza NP protein, interfere with NP self-oligomerization, and attenuate vRNP activity. RPS6P3 was shown to bind to host proteins, including cellular enzymes involved in metabolic pathways and cytoskeletal proteins [36]. K63-linked ubiquitination of RIG-I is an important step that promotes MAVS activation and IFN expression [8,11,53], and lncRNAs are able to facilitate the RIG-I–TRIM25 interaction and suppress influenza infection [35,36,58]. It was demonstrated that 5S rRNA pseudogene transcripts RNA5SP141 triggered RIG-I activation in the late stages of *Herpesviridae* (HSV-1, Epstein-Barr virus) infection and in the case of IAV replication in the nucleus [64]. It is considered that virus-induced host transcription shutoff blocks the production of RNA5SP141-masking proteins (mitochondrial ribosomal protein L18 and thiosulfate-sulfur transferase), leading to RIG-I activation via the demasking of the lncRNA and IFN production [64].

**Another mechanism of lncRNAs antiviral action is the modulation of IFN and ISG expression through chromatin remodeling.** Using this mechanism, lncRNAs are able to attenuate [37] or facilitate influenza infection [61]. It is suggested that IVRPIE enhances IFN-β and ISG (IRF1, IFIT1, IFIT3, Mx1, ISG15, IFI44L) mRNA levels. IVRPIE interacts with its protein partner–heterogeneous nuclear ribonucleoprotein U (hnRNP U, also known as nuclear scaffold attachment factor A, SAFA), which mediates histone modifications (active mark H3K4me3 and repressive mark H3K27me3) in the promoter region of these genes (Figure 3) [37]. Interestingly, in one study [65], hnRNP U was demonstrated to be a nuclear dsRNA sensor that oligomerized and formed an enhanceosome at the distal enhancer of IFNB1. hnRNP U is required for IRF3 and IRF7 recruitment to the promoter. The multimeric hnRNP U form is implicated in chromatin remodulation from a compact structure to open chromatin that is necessary for gene expression [65,66]. This way, the IVRPIE–hnRNP U complex favors the IFN-dependent response.

**LcnRNAs are able to act as ceRNAs and block the effect of ISG-downregulating miRNAs** [42,43,67], **or exhibit proviral action** [44]. The activity of a miRNA is dependent on the concentration of its target molecules, the number of miRNA-binding elements, and the binding affinity between miRNA and its target RNA transcript [68,69]. Therefore, alteration in the level of some miRNA target may affect the regulation and abundance of its other targets [70]. This indirect interaction between RNA transcripts that contain the same miRNA-binding site resembles competition. Thus, ceRNAs are able to titrate miRNAs away from their natural targets, while the expression of competing RNAs correlates with each other [69]. Notably, the levels of each RNA in a ceRNA pair do not need to be the same, and in several cases non-coding RNA expression is less than that of the coding RNA [68,71,72].

Stoichiometric ratios have a decisive influence on ceRNA-miRNA interaction. For instance, in the case of a large number of shared miRNAs, the effect of ceRNA expression modulation is more distinct; however, a single miRNA may have only a minor effect on its target [68]. It is supposed that miRNA-binding sites are more abundant than the number of corresponding miRNAs [68,69]. Importantly, the concentration of miRNAs has an impact on the number of these sites. It is suggested that in the case of low miRNA expression, miRNAs primarily bind to 8- and 7-mer seed sites, whereas miRNA excess leads to the possibility of interaction with low-affinity 6-mer sites. Thus, the stoichiometric ratio affects the size of the competitor pool [68,72].

Stoichiometric ratio of miRNA to miRNA-binding RNAs also has an effect on RNA crosstalk. When the total number of targets largely exceeds the number of miRNAs, minimal competition between RNAs is observed. In the opposite situation (the excess of miRNAs), the competition is also hampered [71]. The optimal crosstalk between two ceRNAs is expected when the contents of miRNA and the corresponding ceRNAs are close to equimolar [71,72]. Similar to these results, conditions with high miRNA:target ratios are not susceptible to derepression by ceRNA competition [68]. This way, the miRNA:ceRNA stoichiometric ratio is the determining feature and should be taken into account in the study of ceRNA action.

Lnc-ISG20 increases the production of ISG20, a well-known antiviral factor. Notably, it upregulates ISG20 at the protein level but does not influence its mRNA expression. Lnc-ISG20 silencing significantly reduced ISG20 mRNA and protein levels in IAV-infected A549 cells. Unsurprisingly, influenza titer and antigen level (NP and M1) negatively correlated with ISG20 production. This way, lnc-ISG20 inhibited IAV replication through ISG20 upregulation. The molecular mechanism of action is based on lnc-ISG20 interaction with miR-326, forming the miR-326- ISG20 complex, which was demonstrated to inhibit ISG20 production at the mRNA level (Figure 3). In other words, lnc-ISG20 acts as a ceRNA for ISG20 mRNA, binding to free miR-326 and preventing miR-326 interaction with ISG20 3′ UTR [43]. IFITM proteins are also regarded to be canonical antiviral factors. A lncRNA, IFITM4P, was identified as a target of miR-24-3p, which was shown to repress IFITM1-3 mRNA. Consequently, IFITM4P overexpression could stimulate IFITM1-3 production. IFITM1 and IFITM3 silencing lowered the IFITM4P level, while IFITM1 or IFITM3 overexpression enhanced IFITM4P. It was concluded that IFITM4P was able to cross-regulate IFITM expression as a ceRNA, interacting with miR-24-3p and blocking its inhibitory action (Figure 3) [42]. SARS-CoV-2 and influenza can stimulate lncRNA-34087.27 expression that also can serve as a ceRNA to stabilize IRF1 mRNA by dampening the level of unbound miR-302b-3p [67].

**Several lncRNAs were shown to positively regulate IFN-dependent signaling.** LncRNA-155 exerts protective action and affects type I IFN-dependent pathways [45,46]. It was revealed that lncRNA-155 knockdown was associated with decreased IFN-β and MxA expression. *In vivo* experiments confirmed the antiviral role of lncRNA-155, as mice with deficiency of the gene were characterized by higher lethality, morbidity, severe interstitial pneumonia with lymphocytic infiltration, elevated viral titer, and viral RNA level in the lungs. LncRNA-155 knockout also led to weakened expression of IFN-β, IFN-γ, and ISG (Mx1, ISG15, OAS3, and IFITM3), as well as IL1β, IL6, IL28, TNF-α, and IRF3 in response to IAV infection [45]. The mechanism of lncRNA-155 action is based on downregulation of PTP1B, which is regarded to be a negative regulator of type I IFN-dependent signaling pathways [45]. PTP1B was shown to negatively regulate IFN-mediated immune response against pulmonary bacterial infection and dephosphorylate JAK2 and Tyk2, as well as STAT3. PTP1B deficiency is associated with ISRE activation, cytokine, chemokine, and IFN-β production, elevated IRF7 expression and activation [73]. Also, PTP1B is considered to be a molecular target of miR-744 that is responsible for upregulating type I IFNs [74]. LncRNA-155 comprises miR-155 that exhibits distinct antiviral action. In mice, pre-miR155 is generated via hydrolysis of the lncRNA-155 by Drosha RNase III, and mature 22-nucleotide miR-155 is cleaved by Dicer RNase III action. In the subsequent study [46], it was demonstrated that lncRNA-155 knockout mice had higher NP mRNA and protein levels, higher viral loads, more severe lung inflammation, and increased morbidity and mortality than mice deficient in the miR-155-encoding fragment (19-bp deletion targeting the pre-miR-155 sequence that prevents its processing and deletes three critically functional nucleotides in the mature miR-155). Also, lncRNA-155 promoted STAT1 phosphorylation, and lncRNA-155-deficient mice had lower levels of IRF3 activation and Ifnb expression than miR-155-deficient animals. The results underlie the primary role of the lncRNA in the combat against influenza infection. The authors suggest that miR-155 alone mainly affects the stages that are downstream of IFN production, primarily targeting SOCS1 and blocking its inhibitory action on JAK/STAT signaling, while full-length lncRNA-155 additionally stimulates innate immune signaling that governs IFN expression (Figure 3) [46].

USP30-AS1 is one of the most universally upregulated lncRNAs in the case of IAV infection. In IAV-infected USP30-AS1 knockout A549 cells, increased viral titer, enhanced production of influenza proteins, and promoted IAV reproduction were associated with a pronounced inflammatory response (elevated IL6 family cytokines, chemokines, guanylate-binding proteins, and ISG15), activation of the complement system and coagulation, and inhibition of cellular biosynthesis. IFN-γ or Poly(I:C) stimulation of USP30-AS-deficient cells resulted in an exacerbated inflammatory response. Consequently, USP30-AS1 can be regarded as an anti-inflammatory modulator of the innate immune response [56].

### 5.3. Proviral lncRNAs

**One of the viral strategies to evade the IFN-dependent response is blocking of IRF activation.** LncRNA THRIL is expressed upon viral invasion and impairs IRF3 activation. THRIL action is associated with reduced IFNB and IFNL mRNA expression and diminished IFN-β secretion, as well as ISG downregulation (OAS1, OAS2, ISG15, IFITM3). Conversely, THRIL knockdown led to increased IFN expression. The lncRNA also inhibited the activity of the IFN-β promoter and IRF3 phosphorylation during viral infection (Figure 2); however, the precise molecular mechanisms were not elucidated [39]. It was shown that THRIL was also required for TNF-α expression induction. THRIL interacts with hnRNPL, and the resulting complex binds to the TNF-α promotor [75]. Moreover, THRIL was demonstrated to be increased in the serum of acute lung injury patients, and its expression positively correlated with elevated inflammatory cytokine levels. In A549 cells, THRIL silencing attenuated TNF-α, IL1β, IL17, and IL6 secretion and decreased the expression of autophagy markers ATG7 and Beclin1 [76]. This way, THRIL alleviates immune response at the stage of IFN and cytokine induction [39].

**Several lncRNAs were shown to exert pro-influenza action via blocking of IFN-β or ISRE promoter activation.** Lnc-MxA inhibits the activation of the IFN-β promoter and downregulates IFNB and ISGs (MxA, IFITM1, IFITM3, ISG15) expression at the mRNA level. Conversely, lnc-MxA knockdown led to increased IFN-β and ISG expression in response to IAV or SeV. It was demonstrated that lnc-MxA is able to form a triplex with the purine-rich region of the IFN-β promoter, which partially overlaps the IRF3 binding site (three nucleotide AAG overlap) and is located near the NF-κB binding site, thus suppressing IFN-β-mediated immune response (Figure 2). Interestingly, the lncRNA did not affect IAV replication in cells with IFNAR1 knockout [38].

TSPOAP1-AS1 knockdown resulted in decreased IAV M-gene expression and M-protein production, as well as reduced viral titer. The key feature of TSPOAP1-AS1 proviral action is its dampening of IAV-induced ISRE promotor activation and ISG (IFIT1, IFITM3, OASL, ISG20) expression (Figure 3), albeit TSPOAP1-AS1 was also known to diminish IFNB expression [40]. In the absence of infection, NRAV negatively regulates ISG expression. After virus detection, NRAV downregulation promotes rapid accumulation of the antiviral factors. NRAV overexpression was associated with downregulation of multiple antiviral ISGs (IFIT2, IFIT3, IFITM3, OASL, MxA) in A549 cells. NRAV inhibited IFN-β- or IFN-λ-induced MxA expression but did not affect total cytokine production or JAK/STAT1 signaling. NRAV blocked promoter activity and the initial transcription of several ISGs (for instance, MxA and IFITM3) through regulation of histone modification (active mark H3K4me3 and repressive mark H3K27me3). It was shown that NRAV interacted with transcription factor zonula occludens 1 (ZO-1)-associated nucleic acid binding protein (ZONAB). The MxA promoter region contains a ZONAB binding sequence, indicating that ZONAB may be involved in MxA expression regulation (Figure 3). ZONAB can upregulate several chromatin remodeling components (histone H4 and high mobility group B1 protein) that recruit core histone-modifying enzymes to DNA. Moreover, transgenic mice expressing human NRAV and infected by IAV were characterized by increased viral load, severe lung inflammation, elevated morbidity, and 100% mortality, whereas about 60% of wild-type littermates have survived [61].

**Additionally, direct influence on immune gene expression, lncRNAs are able to interact with miRNAs that are responsible for the fine-tuning of IFN expression.** Lnc-AROD plays a proviral role during IAV replication and affects influenza infection in C57BL/6 mice. The expression of lnc-AROD led to increased weight loss, elevated mortality, and heightened viral RNA and titer as compared to the reference GFP-overexpressing mice. Lnc-AROD-overexpressing mice were characterized by severe lymphocytic infiltration, a reduction in the alveolar airspace, thickening of the alveolar wall, and an alveolar cavity filled with inflammatory cells in the lungs. Mechanistically, Lnc-AROD expression dampened IAV-induced IFNB, ISG15, and MxA mRNA expression in A549 cells as well as in mouse lungs, albeit it did not affect TBK1 and IRF3 expression. It was revealed that lnc-AROD sponges with miR-324-5p and blocks its activity (Figure 2). In turn, miR-324-5p has a complementary sequence with the 3′-UTR of CUE Domain Containing 2 (CUEDC2) and inhibits CUEDC2 protein production. This way, lnc-AROD weakens miRNA activity and upregulates CUEDC2 level. Elevated CUEDC2 inversely correlates with IFNB, ISG15, and MxA expression and promotes IAV reproduction [44]. CUEDC2 is considered to negatively regulate the JAK-STAT pathway [2].

**Some LncRNAs were demonstrated to contain small open reading frames and encode functional peptides.** For instance, lncRNA PCBP1-AS1 encodes a 110-amino acid small protein called PCBP1-AS1-encoded small protein (PESP). As PCBP1-AS1, PESP expression is upregulated during influenza infection in A549 cells and in response to IFN-β or IFN-λ1 stimulation. IFNAR1 silencing led to weakened PESP production [48]. PCBP1-AS1 facilitated IAV replication, whereas silencing of PCBP1-AS1 or the PESP-coding region hindered viral infection. It was demonstrated that precise PESP production causes alleviated IAV reproduction, as cells with knockout of the PESP-coding region had a significantly lower susceptibility to the infection. Transfection of PCBP1-AS1-deficient cells with PESP-expressing plasmids not only restored but even enhanced IAV replication. Repressed IAV replication was exhibited in 293T cells that overexpress the PCBP1-AS1-delPESP plasmid (PCBP1-AS1 lacking the coding region of PESP) or mutant PESP. The proviral effects of PESP expression are associated with its ability to elevate ATG7 expression and facilitate IAV-induced autophagy (Figure 2) [48]. IAV is known to utilize autophagy to evade the host’s immune response, and ATG7 is regarded to be a distinct proviral factor. ATG7 knockdown caused increased IAV-induced IFNB and IFNL expression. ATG7 diminished IFN response *in vitro* and *in vivo* through an autophagy-independent mechanism underlying ATG7-mediated IRF3 inactivation (Figure 2) [60].

### 5.4. LncRNAs in the Human Genome and Their Conservation in Evolution

It is supposed that IFNs initially appeared in evolution in jawed vertebrates from a common ancestor with interleukins [77], and the complex multiplayer IFN signaling system evolved in amniotes (especially in birds and mammals). IFN-related lncRNAs co-evolved with the signaling system, so their conservation in evolution may yield additional information on their interplay.

To assess this assumption, we reviewed the genomic loci that give rise to the considered lncRNAs in the GRCh3/hg38 human genome assembly. The genes were reviewed manually using the UCSC Genome Browser (queried in November 2025). The sequences that were not annotated in the GRCh3/hg38 assembly were located using nucleotide Blast (NCBI, USA) and the published sequences of lncRNAs, if available. Conservation in evolution, promoter analysis, and the presence of potential regulatory sites were assessed by USCS browser tools PhyloP, ENCODE cCREs, and JASPAR Core 2024, respectively. The results are presented in Table 2. It should be noted that conservation in evolution should be treated with caution, as the alignment of non-translated sequences in distant genomes may be ambiguous.

The analyzed lncRNAs fall into different classes. There are antisense RNAs UPS30-AS1, THRIL, and lnc-MxA, whose mature sequences overlap with exons of the protein-coding genes. Other lncRNAs are intronic (ISR, ISG20); they overlap with coding RNAs at the primary transcript level, but their exons do not overlap. A group of lncRNA genes (TSPOAP-AS1, NRAV, IVRPIE, PCBP1-AS1, and PSMB8-AS1) are located close to protein-coding genes (TSPOAP, DYNLL1, TANK, PCBP1, and PSMB8, respectively). Such lncRNAs are known as bidirectional, as their promoter may potentially overlap with the mRNA promoter, resulting in a bidirectional promoter and correlated expression of the lncRNA and the respective mRNA [78]. The primary transcript of TSPOAP-AS1 also overlaps with mRNAs of the RNF43 and SUPT4H1 genes, making it also an antisense RNA. There are also intergenic RNAs (lncRNA-155, HCG4, lnc-AROD, LINC02574, VILMIR), which are not clearly co-located with some protein-coding genes.

The analysis of potential promoter regulatory sequences revealed the presence of a well-conserved IRF2 site in the promoter of CARINH. Other regulatory elements were not so obvious and require further study. Some annotated lncRNA loci did not contain traits of promoters or enhancers. Some of these lncRNAs may be products of longer RNA products (IVRPIE, lnc-ISG20, IFITM4P), but unconventional initiation mechanisms cannot be ruled out.

As it was discussed above, the lncRNAs utilize diverse mechanisms to regulate IFN-dependent response. However, several common regulatory mechanisms of lncRNAs cannot be ruled out. Short guanine-rich sequences play a significant role in the formation of stable non-canonical structures, such as G-quadruplexes. A canonical G-quadruplex sequence has three or four consecutive guanines separated by loops, with the loop length ranging from one to seven nucleotides (GGGN_1–7_GGGN_1–7_GGGN_1–7_GGG) [79]. G-quadruplex sequences may be located in functionally important regions, including promoters or untranslated regions, and are considered to be highly conserved [79,80]. These specific regions may interact with RNA-binding proteins containing arginine-glycine repeats or zinc finger domains [81,82,83]. These factors involve lncRNA in the regulation of transcription and translation [83,84]. Among IFN-response modulating lncRNA could be several highly conserved guanine-rich motifs that may serve as protein binding sites. For instance, using MEME Suite 5.5.9 Version [85], we noted that the guanine-rich motif GGAGG[CAT][GTC]GAGG[CT][ATG]G[GC] is typical for the majority of IFN-modulating lncRNAs (Figure S1). Serine/Arginine-rich Splicing Factors 1 (SRSF1) was shown to directly bind to a lncRNA that is implicated in pulmonary fibrosis pathogenesis [82]. We hypothesize that this lncRNA can function as a regulatory molecule, directly interacting with the components of the spliceosome and participating in the control of alternative splicing of target mRNAs. Further bioinformatic analysis is required for searching the specific regulatory sequences and lncRNA protein partners, as well as the miRNA-encoding capacity of these lncRNAs.

The evolutionary conservation of whole lncRNAs may be characterized as relatively low. This is not very surprising: in the evolution of lncRNAs, the RNA structure, rather than the sequence, may be preserved (structure conservation in antiviral lncRNAs is not reviewed here due to the scarcity of published data). Naturally, segments of antisense RNAs that overlap the coding sequence of mRNAs are highly conserved among mammals. The same applies to the region of lncRNA-155, which is the precursor of conservative micro-RNA miR-155 (see Section 5.2 above) [45]. In other parts, homologs of human sequences were readily found in primates and, with some variations, in rodents, but for more distant species the homology was not clear. However, it should be noted that the genes of many of the analyzed lncRNAs contain isolated 50–100 nucleotide loci with above-average sequence conservation in mammals and, in some cases, in vertebrates. These loci do not readily correlate with lncRNA exons or JASPAR regulatory sites. The relation between these loci and the biogenesis, structure, or function of lncRNA is not clear at present.

**Table 2:** Considered LncRNAs in human genome and their conservation in evolution.

lncRNA (Locus Name or Genomic Coordinates) ^1^	Class	Annotated RNA Variants ^2^	Exons ^3^	Associated Genes and Sequences	Promoter ^5^	Predicted Regulatory Elements (CREs) ^6^	Conserved Sequence Elements; Conservation Range (MultiZ)
HCG4	intergenic	1	ND	in MHC gene complex; antisense to HLA-V (Major Histocompatibility Complex, Class I, V (Pseudogene)), inside of HLA-F-AS1 RNA intron 2 (↑↑) ^4^	Typical		Not revealed
IFITM4P	pseudogene	1	ND	in MHC gene complex; inside intron 2 of HLA-F-AS1 RNA intron 2 (↑↑), ~40 kb from HCG4, contains interferon-induced transmembrane protein pseudogene;	Not predicted (may originate from HLA-F-AS1 RNA)		Described in human genome only
ISR (MN397203)	intronic (interleaved exons)	1	2	BAHCC1 chromatin-associated protein (↑↓, no exon overlap) overlaps MIR4740 (↑↑)	Not predicted		3–4 short regions (2 in exon 1); mammals
IVRPIE (chr2: 161135501-161136807)	bidirectional with TANK?	1	ND	TANK is TRAF Family Member Associated NFKB Activator (↑↓, promotor overlap?); located inside intron 2 of TANK-AS1 RNA (↑↑);	Not predicted (may originate from TANK-AS1 RNA)		5′-terminus + ~50 bp region; placental mammals
LINC02574 (LINC02574)	intergenic	1	3	overlaps 5′-UTR of IFI6 (interferon alpha inducible protein 6) mRNA	Inner	CTCF site, conserved in mammals	3–5 intronic regions; placental mammals
lnc-ISG20-1 (chr15: 88621381-88626179)	intronic	-	2	inside the intron of AEN (Apoptosis Enhancing Nuclease) gene (↑↑), potential target gene (ISG20, Interferon Stimulated Exonuclease Gene 20) is within 10 kb range	Typical (may be promoter of AEN gene)	Weak TP53 sites	Not revealed
lnc-ISG20-2(chr15: 88717277-88717721)	intergenic	-	1		Not predicted		3~100 bp ranges; mammals
lncRNA-155 (MIR155HG)	intergenic	6	5	contains MIR155–miR-155 microRNA sequence (↑↑)	Inner		MIR155 subrange, parts of exons 2 and 3 and intron 2; placental mammals
RPS6P3	intergenic, retrogene	1	1	Comprises Ribosomal Protein S6 Pseudogene 3	Not predicted	IRF1 site	Remnant of S6 protein coding sequence; primates
USP30-AS1	antisense	1	2	antisense to coding exon 1 of USP30 (Ubiquitin Specific Peptidase 30)	Typical	IRF site STAT1:STAT2 sites	overlap with coding region of USP30; mammals
GAPLINC	intergenic	4	3–4	no apparent interaction	Typical, conserved in mammals	2 IRF sites LEF1 site FOXO1 site; conserved in mammals	Enhancer (possibly); mammals
lnc-AROD (LNCAROD)	intergenic	>10	7	promoter is adjacent to MBL2 promoter (↑↓), exons interleave with some poorly annotated RNAs (↑↓)	Typical	HSF (heat shock) sites	5~50–100 bp intronic regions; mammals
lnc-MxA (MX1-AS1)	antisense	1	3	antisense to exons 10 and 12 of Mx1 gene (Interferon-Regulated Resistance GTP-Binding Protein MxA)	Not predicted		regions antisense to Mx1 coding sequence; mammals
NRAV	bidirectional with DYNLL1	2	2	DYNLL1 is Dynein Light Chain LC8-Type 1 (↑↓, transcripts do not overlap, promoters overlap)	Typical, moderately conserved in mammals		start of exon 1; mammals
PCBP1-AS1	bidirectional with PCBP1, antisense?	>20	11	PCBP1 (Poly(rC) Binding Protein 1) retrogene (↑↓, promoter overlap); some RNA variants may overlap PCBP1	Inner		2 intronic ~100 bp ranges; placental mammals
PSMB8-AS1	bidirectional or antisense with *PSMB8*	4	2–3	antisense to coding exon 1 of PSMB8 mRNA variant (proteasome 20S subunit beta 8), overlaps coding exon 1 of TAP1	Typical, overlaps promoter of *PSMB8*, conserved in mammals	Weak IRF3, conserved in mammals	overlap with coding region of TAP1; vertebrates
TSPOAP1-AS1	bidirectional with TSOAP1	>10	3	antisense to coding exon 2 of RNF43, RING Finger Protein 43; TSOAP1, Benzodiazapine Receptor Peripheral-Associated Protein 1 (↑↓, transcripts do not overlap, promoters overlap); SUPT4H1, DSIF Elongation Factor Subunit, in intron 1 (↑↓); variants may overlap microRNA gene MIR142HG (↑↓)	Inner	RFX1 site, conserved in mammals	overlap with coding region of RNF43; vertebrates
THRIL	anisense	1	1	Antisense to 3′-UTR of BRI3BP mRNA (BRI3-Binding Protein, Cervical Cancer Oncogene Binding Protein)	Not predicted		~50 pb range; amniotes
VILMIR	intergenic	>10	3	Exon 3 in humans contains an unspecified pseudogene RP11-640	Not predicted		exon 1; primates

Comments: ^1^ The locus name in the GRCh3/hg38 human genome assembly is given (in parentheses) only if it is different from the lncRNA conventional name; for lncRNAs that have no records in GRCh3/hg38, gene coordinates are given. ^2^ The number of the reported RNA variants (including splicing variants) in the present databases is indicated. ^3^ Exon count is given for the reference gene in the GRCh3/hg38 assembly; possible transcript variants and putative alternative splicing variants are not listed. ND–no data. ^4^ Symbol ↑↑ indicates that the lncRNA is transcribed in the same direction as the referenced gene; ↑↓ indicates transcription in the opposite direction (may be a prerequisite for RNA interference). ^5^ Promoters are specified using ENCODE4; the predicted promoter is termed typical if it is located upstream of the lncRNA locus and is accompanied by cis-regulating elements (CREs); it is termed inner if the predicted promoter is located inside the reference lncRNA locus. If the promoter is well conserved, a respective statement is added. Not predicted–no promoters or CREs were predicted near the putative translation initiation site. ^6^ Prominent JASPAR-predicted sites with high scores are listed, which were conserved at least in primates/rodents and/or were related to interferon signaling.

## 6. Conclusions and Perspectives

Nowadays, it is known that many lncRNAs are able to control IFN-dependent responses and the susceptibility to influenza infection. LncRNAs can govern either systemic IFN response in white blood cells or antiviral IFN-dependent response in respiratory epithelial cells. Generally, the lncRNAs are induced by RNA-containing viruses, such as IAV, VSV, or SeV, as well as poly(I:C) or IFN-β treatment in a RIG-I and NF-κB-dependent manner. Thus, lncRNAs are primarily regulated through the RIG-I/IFN-β/IFNAR1 pathway and can be considered as ISGs with either antiviral or proviral functions. Although the features of lncRNA action are not known in all cases, it is possible to determine some key mechanisms of their influence on IFN and ISG expression (Figure 2 and Figure 3). One of lncRNA’s functions is to limit IFN and ISG expression, especially in the late stages of infection, and several viruses are able to utilize lncRNAs to evade the innate immune response. On the contrary, a number of lncRNAs stimulate RIG-I activation, IRF phosphorylation, and IFN and ISG expression. Also, we reviewed the genes of lncRNAs that are involved in IFN expression regulation and the evolutionary conservation of these regulatory molecules. The lncRNAs are spread among different classes: antisense, bidirectional, intronic, or intergenic RNAs. The evolutionary conservation of analyzed lncRNAs was below the expectations and may be characterized as relatively low. Homologs of human sequences were readily found in primates and, with some variations, in rodents, but for more distant species the homology is not clear.

Research of lncRNAs is an extremely promising scientific area, and it is expected that a significant number of new members of this class of biomolecules will be discovered in the nearest future. These studies may result in the development of new approaches to influenza infection treatment, as well as advanced understanding of the disease pathogenesis. Further bioinformatical analysis, including an in-depth search of common regulatory sequences, miRNA encoding capacity, lncRNA spatial structure, and finding their protein partners, could reveal putative common mechanisms of lncRNA action.

## Data Availability

No new data were created or analyzed in this study. Data sharing is not applicable to this article.

## Supplementary Materials

The following supporting information can be downloaded at: https://www.mdpi.com/article/10.3390/v18020231/s1, Figure S1: Guanine-rich conservative motif.

## Abbreviations

The following abbreviations are used in this manuscript:

AdV	Adenovirus
CARD	Caspase activation and recruitment domain
ceRNA	competing endogenous RNA
CUEDC2	CUE domain containing protein 2
GAF	γ-activated factor
GAS	γ-activated sequence
HSV	Herpes simplex virus
hnRNP	Heterogeneous nuclear ribonucleoprotein
SAFA	Scaffold attachment factor A
IAV	Influenza A virus
IFN	Interferon
IFNAR	Type I interferon-receptor
IFIT	Interferon-induced proteins with tetratricopeptide repeats
IFITM	Interferon-induced transmembrane protein
IFNLR	Type III interferon receptor
IKK-ε	Inhibitor of NF-κB kinase-ε
IRF	Interferon regulatory factor
ISG	Interferon-stimulated gene
ISGF3	Interferon-stimulated gene factor 3
ISRE	Interferon-stimulated response element
JAK	Janus kinase
LPS	Lipopolysaccharide
MDRV	Muscovy duck reovirus
lncRNA	Long noncoding RNAs
MAVS	Mitochondrial antiviral signaling protein
MDA5	Melanoma Differentiation-Associated protein 5
MxA	Myxovirus resistance protein
NF-κB	Nuclear factor κB
NP	Nucleoprotein
NS1	Non-structural protein 1
OAS	2′-5′-oligoadenylate synthetase
PAMP	Pathogen-associated molecular pattern
PESP	PCBP1-AS1-encoded small protein
PRV	Pseudorabies virus
Poly(I:C)	Polyinosinic:polycytidylic acid-
PRR	Pattern recognition receptor
PTP1B	Protein-tyrosine phosphatase 1B
RIG-I	Retinoic acid-inducible gene 1
RSV	Respiratory syncytial virus
SeV	Sendai virus
SOCS	Suppressor of cytokine signaling
STAT	Signal transducer and activator of transcription
TLR	Toll-like receptor
TRIM25	Tripartite motif-containing protein 25
TYK	Tyrosine kinase
USP18	Ubiquitin specific peptidase 18
VSV	Vesicular stomatitis virus
