# Supplementary material for: Long Non-Coding RNAs Can Govern the Antiviral Immune Response Through Interferon-Mediated Mechanisms in Respiratory Tract
Source: Viruses. 2026 Feb 12;18(2):231. doi: 10.3390/v18020231 (PMC12944882; doi:10.3390/v18020231)
Supplement: Supplementary file 1 [file viruses-18-00231-s001.zip › viruses-4100970-supplementary.pdf]

Supplementary File

We analyzed 20 lncRNA sequences using the MEME (Multiple EM for Motif Elicitation) suite (version 5.5.9) (Timothy L. Bailey, 2015). The analysis was performed with the following key parameters: motif distribution, number of motifs, motif width, statistical significance. The command line that was used:

```
~$ meme all_lncRNA.fa -o meme_results -dna -mod anr -nmotifs 20 -minw 6 -maxw 15 -revcomp
```

MEME generated output files containing the position-specific probability matrices for each discovered motif, along with E-values, logos, and site location in the input sequences.

We identified 8 highly conserved motifs. Among these motifs, we found a conservative ($3.3e^{-036}$) 15 nucleotide motif (GGAGG[CAT][GTC]GAGG[CT][ATG]G[GC]) that is rich in guanine (Figure S1). The motif presents in 17 of the studied lncRNAs (ISG-20, LINC0257, NRAV, THRIL, USP30-AS1, PCBP1-AS1, HCG4, ENST00000613639.3, CARINH, PSMB8-AS1, MIR155HG, TSPOAP1-AS1, IFITM4P, lnc-AROD, and CHROMR). Thus, the motif was found in 85% of the analyzed lncRNAs, suggesting it is a highly conserved element.

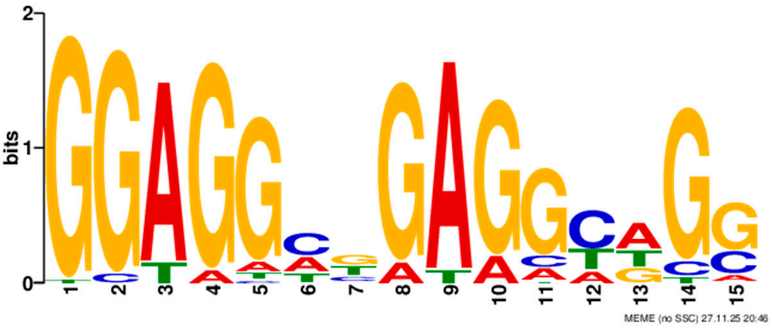

Figure S1 – Guanine-rich conservative motif
